# Importance of Return to Usual and School Activities After Social Isolation in Recovering Vitamin D Concentrations, Physical Fitness, and Motor Performance in Adolescents

**DOI:** 10.3390/ijerph21111494

**Published:** 2024-11-10

**Authors:** Frederico Bento de Moraes, Maiara Cristina Tadiotto, Brenda Lenardt, Jorge Mota, Oslei de Matos, Neiva Leite

**Affiliations:** 1 Department of Physical Education, Federal University of Paraná, Curitiba 81531-980, PR, Brazil; 2 Densitometry Research Laboratory, Postgraduate Program in Physical Education and Health, Technological Federal University of Paraná, Curitiba 81531-980, PR, Brazil; 3 Center for Research in Physical Activity, Health and Leisure (CIAFEL)—Faculty of Sports, University of Porto (FADEUP), 4200-450 Porto, Portugal

**Keywords:** youths, motor competence, COVID-19, physical conditioning

## Abstract

This study aimed to observe adolescents’ changes and individual responses regarding the anthropometry, cardiometabolic profile, vitamin D concentrations, physical fitness, and motor competence upon immediate return and three months of school activities after lockdown. Methods: The study included 28 adolescents (14.8 ± 0.8 years) of both sexes. Anthropometric measures, body composition, cardiometabolic parameters, physical fitness, and motor competence were assessed. A paired *t*-test was used to compare the frequencies of respondents and the effect size of the results, considering significant *p* < 0.05. Results: After three months of school activities, adolescents changed their body composition, reducing % fat mass (*p* = 0.008) and increasing fat-free mass (*p* = 0.008). In terms of physical fitness, there was increased abdominal resistance (*p* < 0.001; ES = −0.42) and motor performance, with reduction in supine-to-stand test time (*p* < 0.001; ES = 0.53). There were very beneficial effects in reducing resting heart rate (*p* ≤ 0.001; ES = 0.61) and increasing vitamin D concentrations (*p* < 0.001; ES = −0.61). After three months of a school routine, the proportions of change in respondents were similar between girls and boys and eutrophic individuals and overweight individuals. Conclusions: Returning to school activities after lockdown was important for the recovery of vitamin D concentrations, physical fitness, and motor competence, whose responses were independent of the level of adiposity and sex of the adolescents.

## 1. Introduction

Schools are important settings for the cognitive and social development of children and adolescents, whose activities in physical education classes play a fundamental role in motor development and physical fitness [1]. However, in-person school activities were banned for about two years in Brazil, due to the restrictions and lockdown caused by the coronavirus-19 (COVID-19) pandemic, officially declared in March 2020 [2]. Measures taken to control the spread of the virus, including lockdowns and school closures, had profound implications on adolescents’ lives, affecting not only their educational environment but also their physical and mental health [3]. The confinement of families indoors led to reduced sun exposure, which negatively impacted vitamin D (25(OH)D) concentrations. This vitamin is crucial for bone development and growth [4].

School closures led to significant changes in adolescents’ routines due to social isolation. Although some access to outdoor activities was permitted, restrictions varied throughout the pandemic, with intermittent measures limiting access to public spaces like parks and beaches, impacting opportunities for outdoor play and physical activities. There was a significant reduction in physical activities, an interruption of structured exercise routines, an increase in screen time for remote classes, and changes in eating and sleeping habits [5,6]. These factors contributed to conditions that directly impacted young people’s physical and motor fitness, including decreased cardiorespiratory endurance, muscle strength, flexibility, motor coordination [7], and body mass gain [8,9].

Pre-pandemic studies have shown that physical and motor fitness in adolescence influences sports performance and is directly related to cardiometabolic health, mental health, and overall quality of life [10,11]. The sudden reduction in physical activity during lockdown likely affected adolescents’ physical fitness, motor development, and 25(OH)D concentrations. Therefore, understanding the pandemic’s impact on adolescent health and the effects of resuming school activities is essential to inform recovery strategies and public health initiatives.

Some studies have found that school closure and social distancing measures have negatively affected adolescents’ physical activity and well-being [12,13] and showed accelerated changes in the cortical thinning of the brain, mainly due to the lack of social interaction during isolation [14]. However, they did not examine whether favorable changes in physical and motor fitness occurred after returning to school activities. Therefore, this research sought to fill this gap in the literature by providing a longitudinal analysis of adolescents’ physical and motor fitness after returning to usual pre-pandemic activities, following the end of restrictions. The study aimed to verify anthropometric changes, the cardiometabolic profile, vitamin D concentrations, physical fitness, and motor competence upon immediate return and three months of school activities after lockdown, and analyze individual responses of boys and girls, as well as eutrophic and overweight adolescents.

## 2. Materials and Methods

This observational study monitored 28 adolescents (32.1% girls and 67.8% boys), aged 13 to 16 years, as they returned to school activities. A total of 36 adolescents were initially invited to participate, but only 28 completed both the baseline assessment and the three-month follow-up after the return to classes. The study was conducted by the Quality-of-Life Center of the Federal University of Paraná (NQV-UFPR). The participants and their parents/guardians received a detailed explanation about the evaluations; after agreeing to the study procedures, they signed an informed consent form, and the adolescents signed an informed assent form. The research followed the guidelines and standards for regulating human research, and the procedures with the students were previously approved by the Ethics Committee of the UniDomBosco University Center (CAAE 62963916.0.0000.5223).

The adolescents were recruited through invitations to a public school in Curitiba, Paraná, Brazil. The inclusion criteria were (a) being interested and participating in all assessments; and (b) being 13 to 16 years old. The exclusion criteria were (a) participants with muscle injuries or any other contraindication to taking the tests; (b) taking medications that could interfere with the test results; and (c) being involved in any regular physical activity other than regular physical education classes at school. School physical activities followed the school curriculum, with two physical education classes a week focusing on basic sports, below the physical activity recommendations for adolescents.

Participants’ anthropometric measures, physical fitness, and motor development were assessed in the initial phase and after three months at the school, following the procedures described below. Body mass (BM) (kg) was measured on a Welmy^®^ model W200/50^®^ digital scale, height (cm) was measured on an Avanutri^®^ portable stadiometer, and BMI-z was calculated using the WHO Anthro Plus^®^ program [15]. Anthropometric measures were taken as described in [16]. The sitting height was measured using a 50 cm bench attached to a stadiometer, while leg length was calculated by subtracting the sitting height from the total height. Waist circumference (WC) was assessed with an anthropometric tape applied to the skin in the area between the ribs and the iliac crest. The waist-to-height ratio (WHtR) was calculated by dividing WC (cm) by height (cm).

Body composition was assessed by dual-energy X-ray absorptiometry (DXA), Hologic Discovery A (Mississauga, ON, Canada), to estimate the fat-free mass (FFM), fat mass (FM), and fat mass percentage (%FM). Quality Control was verified daily before each assessment [17].

Systolic and diastolic blood pressures were measured using a previously calibrated mercury sphygmomanometer with an appropriately sized cuff. Measurements were taken after 10 min of rest, with the adolescent sitting and their right arm supported at heart level. A specialized team collected blood samples in the morning after 12 h of fasting. The enzymatic colorimetric method was used to measure total cholesterol and triglycerides. The chemiluminescence method was used to determine glucose, and the electrochemiluminescence method determined 25(OH)D.

Somatic maturation was assessed by a mathematical model based on anthropometry, age, and sex, determined by predictive equations for peak growth velocity [18]. The age of peak height velocity was calculated by subtracting the maturity offset from the chronological age.

Motor performance and competence were assessed by the supine-to-stand (STS) test, based on the protocols suggested by Vansant [19]. The participant was asked to lie down in a supine position on a flat surface, with the upper limbs extended along the body and lower limbs. They were instructed to stand up as quickly as possible in any way they wanted after an audible command and maintain the standing position. No demonstration was performed so as not to influence their movement patterns. Three attempts were performed—the first one for familiarization with the test and the other two filmed in the sagittal plane with a digital camera for a subsequent analysis using the Kinovea^®^ program. The shortest execution time was used to determine the STS_TIME_ performance. The STS_MC_ was evaluated based on the motor patterns to stand up from the floor, classified by the sequences of movements of the upper and lower limbs and body axis [19].

Cardiorespiratory fitness was assessed using the shuttle run test [20] at the school’s sports hall. The protocol consists of multiple progressive stages of running with increasing intensity, in which participants must move between two markers 20 m apart at the pace set by an audible signal. The initial speed was 8.5 km/h^−1^, increasing by 0.5 km/h^−1^ per stage. A heart rate monitor Polar^®^ A300 (Polar Electro, Kempele, Finland) measured maximum heart rate, and the test was only interrupted at the participant’s request or when they did not reach the line marked for the audible signal during two consecutive laps. The adolescents were previously familiarized with the test, and later cardiorespiratory fitness was estimated by a specific equation for adolescents proposed by Menezes-Junior et al. [21].

Upper limb strength was assessed through handgrip testing, obtained with a handheld hydraulic dynamometer Saehan^®^ (Saehan Corporation, Masan, South Korea) whose grip was adjusted for each participant, with the elbow flexed at a 90° angle with the forearm in the neutral position. Participants were instructed to tighten the equipment gradually and continuously until maximum strength was achieved. Two attempts were performed, with a 60 s interval between attempts, and each participant’s highest value was recorded in kilograms. The abdominal resistance assessment considered the maximum number of abdominal crunches performed in one minute [22], and flexibility was determined with Well’s sit-and-reach test [23].

## 3. Statistical Analysis

Data normality was verified using the Kolmogorov–Smirnov test and the assumption of homogeneity of variance was evaluated using Levene’s test. Descriptive statistics were presented using the mean and standard deviation, and differences between return and post-return to school activities were compared using Student’s paired *t*-test and the Wilcoxon test for nonparametric data. Confidence intervals (95%) of the effect size (ES) were calculated to determine the magnitude of the intervention effect. Clinical inference was performed according to the magnitude of the standardized ES, as follows: trivial (≤0.2), possibly beneficial/harmful (ǀ0.20–0.39ǀ), beneficial/harmful (ǀ0.40–0.79ǀ), and very beneficial/harmful (>0.80) [24]. The frequencies of individual responsiveness were calculated based on the theoretical model and the magnitude of the individual effect, dividing the delta values (final value—initial value) by the grouped standard deviation (SD), with the following formula: √((DP_1_^2^ + DP_2_^2^)/2). It verified the frequency of respondents among boys, girls, eutrophic participants, and overweight participants. The cutoff point for effective responsiveness was set at ES ≥ 0.20 or ≤−0.20 [25]. The statistical analysis was performed using SPSS version 26.0, and the significance level was set at *p* ≤ 0.05.

## 4. Results

This observational study monitored 28 adolescents (14.8 ± 0.8 years) in their return to school activities. Table 1 presents the descriptive characteristics and comparisons between the initial assessment (immediate return after removal of social restrictions due to the COVID-19 pandemic) and the reassessment (after three months of usual school activities) and the ES results of the anthropometric variables, body composition, physical fitness, and motor competence. The participants’ anthropometric measures did not change significantly over the period (*p* > 0.05), except for a statistically significant, though trivial, increase in height (*p* < 0.002; ES = 0.00), indicating a small effect size despite statistical significance. Body composition also had a trivial difference, with a reduction in %FM and an increase in FFM (*p* = 0.008: ES = 0.10, and ES = 0.12, respectively). Regarding physical fitness, after three months, there was a possibly beneficial increase in abdominal resistance (*p* < 0.001; ES = 0.42) and an improvement in motor performance with a beneficial reduction in the time taken to perform the STS_TIME_ (*p* < 0.001; ES = −0.53). The other variables were not different (*p* > 0.05).

Table 2 shows the comparisons between the sample’s initial clinical assessment and metabolic profile with the reassessment three months after usual school activities. There was a beneficial reduction in HR_rest_ (*p* ≤ 0.001; ES = 0.61) and a beneficial increase in 25(OH)D (*p* < 0.001; ES = 0.61). The other variables had no differences (*p* > 0.05).

**Figure 1:**
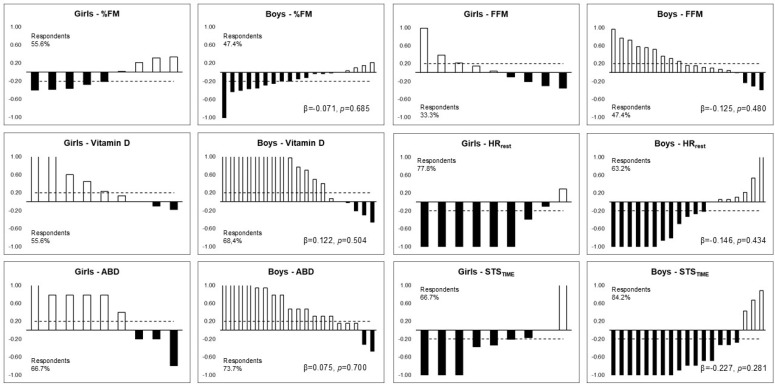
Frequency of respondents in body composition, vitamin D, abdominal strength, resting heart rate, and motor performance after three months of returning to classes among boys and girls.

Figure 1 shows the respondents’ frequency of body composition, 25(OH)D, HR_rest_, abdominal resistance strength, and motor performance among boys and girls. No differences were observed in the frequencies of respondents and non-respondents between the sexes three months after returning to school. The proportions of change in respondents were similar, respectively, for girls and boys, for %FM (55% and 47%), 25(OH)D (55% and 68%), HR_rest_ (77% and 63%), abdominal resistance (66% and 73%), and STS_TIME_ (66% and 84%).

**Figure 2:**
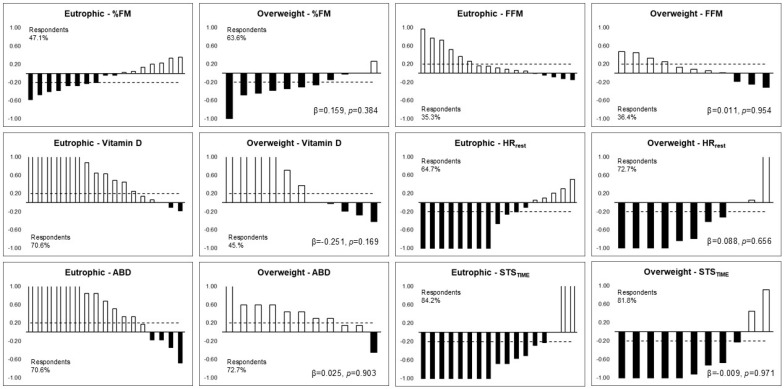
Respondents’ frequency of body composition, vitamin D, abdominal strength, resting heart rate, and motor performance after three months of returning to school among eutrophic adolescents and overweight adolescents.

Figure 2 shows the respondents’ frequency of body composition, 25(OH)D, HR_rest_, abdominal resistance strength, and motor performance between eutrophic individuals and overweight individuals. No differences were observed in the frequencies of respondents and non-respondents between eutrophic individuals and overweight individuals three months after returning to school. The proportions of change in respondents were similar, respectively, for eutrophic and overweight individuals, for %FM (47% and 63%), 25(OH)D (70% and 45%), HR_rest_ (64% and 72%), abdominal resistance (70% and 72%), and STS_TIME_ (84% and 81%).

## 5. Discussion

The main findings of this research suggest an improvement in several health-related factors, such as 25(OH)D concentrations, body composition, physical fitness, and motor performance, three months after the return of usual school activities (after the social isolation to combat the spread of COVID-19). This highlights the role of school activities, especially physical activities and sports, which are very important not only for academic development but also for promoting adolescents’ health and physical well-being.

Although the exact mechanisms by which the resumption of school activities contributes to the recovery of factors such as vitamin D and physical fitness still need further investigation, our results suggest a positive and consistent response among adolescents of different body profiles. The absence of significant differences between genders and levels of adiposity reinforces the effectiveness of resuming school for the health of all adolescents. However, the increase in blood pressure levels observed in some participants requires attention: while regular physical activity tends to benefit the cardiovascular system, factors such as the intensity of the return and lifestyle changes during the pandemic may have influenced these results.

Physical activities decreased and screen time increased among adolescents in social isolation during the pandemic [26], mainly due to the ban on outdoor activities and gyms. The increase in screen time may be related to online remote school activities, which further aggravated an existing problem that corroborates physical inactivity and an increase in sedentary lifestyles. In addition, the use of electronic devices during the day and before bed is a main factor in the development of sleep disorders [27] and may increase the prevalence of obesity and other comorbidities in children and adolescents [28], compromising physical fitness and motor performance [29]. Adolescence is an important phase for motor skill development, influenced by biological and environmental factors [30,31]. The lockdown measures to contain the spread of the virus during the pandemic may have resulted in losses in 25(OH)D concentrations, physical fitness, and motor development of children and adolescents.

The length of the government isolation decrees worldwide hindered not only physical activities but also sunlight exposure, significantly decreasing 25(OH)D concentrations in school-aged children and adolescents [4]. Furthermore, lower 25(OH)D levels were associated with more severe cases of COVID-19 in children and adolescents in a study conducted during the pandemic [32]. The 25(OH)D concentration recommended by the Brazilian Society of Endocrinology and Metabolism (SBEM) is above 20 ng/mL, a desirable value for a healthy population up to 60 years old [33]. Participants in this study presented exactly the desired minimum; assuming the standard deviation, we can consider that some participants were below the recommended 25(OH)D concentration.

However, with the end of restrictions and the return to in-person activities, the concentration of this vitamin improved considerably from the initial assessment. In the study by Bayramoğlu et al. [32], most of the sample of children and adolescents (1 to 18 years old) had a concentration of 25(OH)D below the recommended level even before the pandemic. After dividing them by age groups, the value was even lower in adolescents with a similar age to our study. In addition, the concentration of 25(OH)D decreased by an average of 2.1 ng/mL during the first year of the pandemic, a value below the increase in our participants three months after returning to activities.

A school is an important place for physical activities among adolescents. Therefore, resuming in-person school activities and reopening public and leisure spaces can be directly related to improving a more active and healthy lifestyle [3]. These factors reflect the positive changes found in this study, mainly related to body composition since the increase in energy expenditure provided by the increase in activities after social isolation decreased %FM and increased FFM. In addition, there was a significant increase in abdominal resistance strength and a reduction in basal heart rate, physiological changes related to the increase in physical activity. These findings suggest the importance of physical activities at school and the return to usual daily activities, as they can help improve health by increasing strength and improving the cardiovascular system [10]. Nevertheless, the cardiorespiratory fitness test did not have a significant result, which can be attributed to the short time for the cardiovascular system to adapt, and we do not know whether these participants’ cardiovascular capacities were affected by isolation. The lack of significant findings in cardiorespiratory fitness may also be due to the short duration of the intervention or the moderate intensity of the school-based physical activities, which might not have been sufficient to stimulate substantial cardiovascular adaptations. Additionally, the limitations in frequency (twice a week) could have restricted the potential for noticeable improvements in aerobic capacity.

The development of motor skills appears to have a positive role in preventing unhealthy markers in children [34], and abdominal resistance strength is an important factor in physical fitness for performing motor skills in daily activities such as getting out of bed. Thus, the STS aims to assess the participant getting up from a lying position, an activity that recruits abdominal strength for the initial phase and complete movement. The changes in physical fitness in this research showed motor performance improvements after the return to in-person school activities, with a significant reduction in the time taken to perform the STS_TIME_. This result is reciprocal of the increase in physical fitness and consequent improvement in motor performance proposed in the theoretical model of Stodden et al. [35]. Furthermore, a study evaluating children’s and adolescents’ motor competence found a strong relationship between motor competence and health-related physical fitness variables [36]. The response to resuming pre-pandemic routines with outdoor activities, school activities, and structured physical activities in physical education classes had a combined positive impact on adolescents’ motor performance and functional capacities.

The study analyzed the frequency of responsiveness among boys and girls and eutrophic and overweight adolescents due to the importance of interindividual variability, considering the variables that had changed from the initial assessment when activities resumed. Although no significant frequencies were found, there was a greater proportion of changes in the variables among respondents. The lack of significant frequencies highlights the homogeneity of the group of adolescents in this study, which reduces data variability. Factors such as baseline fitness levels, genetic predispositions, and socio-environmental influences may have contributed to the differences observed in the participants’ responses. Future research should consider controlling these variables to better understand the individual factors that influence responsiveness to physical activity interventions.

This study has some limitations that should be considered, including the small sample size and the selection of adolescents from a single school. Therefore, generalizing the results to a wider population should be carried out with precaution. Another point to be considered is the relatively short duration of the study, only three months, which may not have been enough to fully capture the changes in all variables in question. The lack of data on physical activity outside the school environment and pre-pandemic baseline data also limits the ability to determine whether observed changes are exclusively due to the resumption of school activities or represent a recovery of pre-pandemic levels. However, this study monitored in detail the changes in physical fitness, motor performance, and metabolic analyses after three months of the return to usual daily and school activities in a specific group of adolescents. It is noteworthy that in Brazil, schools were closed for face-to-face activities for two years, due to social isolation in response to the COVID-19 pandemic. This is a factor that may have impaired the performance of the variables studied. Returning to routine activities demonstrated important benefits, regardless of sex or adiposity of teenagers.

This study did not capture data on physical activity outside the school environment, suggesting the need for specific questionnaires in future research. Given the observed short-term improvements, future studies should explore the long-term effects of returning to regular physical activity post-isolation. Longitudinal studies, incorporating detailed data on both school-based and extracurricular physical activities, could provide valuable insights into the sustained impact on adolescent health, including cardiovascular fitness, mental well-being, and social behavior.

## 6. Conclusions

This study provides evidence that the return to usual and school activities after social isolation was important for the development and recovery of physical fitness, motor development, and vitamin D concentration, whose responses were independent of the level of adiposity and sex of the adolescents. Thus, we emphasize the importance of promoting healthy habits at home, in leisure facilities, and especially at school, providing regular physical activity and sun exposure, important factors for the health of students.

## Figures and Tables

**Table 1:** Comparison of immediate return and three months of usual activities on anthropometric variables, body composition, physical fitness, and motor competence.

Variables	Immediate Return	Three Months of Usual Activities	ES	CI	*p*
Anthropometrics and body composition					
Height	1.67 ± 0.09	1.67 ± 0.09	0	T	0.002
BM	61.3 ± 14.0	61.8 ± 13.6	0.04	T	0.281
BMI-z	0.41 ± 1.4	0.38 ± 1.3	−0.02	T	0.31
BMI	21.9 ± 4.8	21.9 ± 4.4	0	T	0.726
WC	73.1 ± 11.3	73.2 ± 10.5	0.01	T	0.982
WHtR	0.43 ± 0.06	0.43 ± 0.06	0	T	0.515
FM_DXA	18.2 ± 8.5	17.7 ± 8.4	−0.06	T	0.115
%FM_DXA	28.7 ± 8.6	27.8 ± 8.7	−0.1	T	0.008
FFM_DXA	43.1 ± 8.3	44.1 ± 8.6	0.12	T	0.008
Physical fitness and motor performance					
FLEX	19.0 ± 12.5	20.1 ± 11.0	0.09	T	0.213
HGR	29.4 ± 6.9	29.1 ± 6.9	−0.04	T	0.904
HGL	27.6 ± 7.3	27.3 ± 7.2	−0.04	T	0.718
ABD	24.2 ± 8.4	27.9 ± 9.4	0.42	B	<0.001
CRF_rel_	38.8 ± 8.1	38.7 ± 8.0	−0.01	T	0.953
CRF_abs_	2.31 ± 0.3	2.34 ± 0.4	0.08	T	0.253
STS_MC_	10.0 ± 2.3	9.9 ± 2.3	−0.04	T	0.858
STS_TIME_	2.45 ± 0.52	2.20 ± 0.41	−0.53	B	<0.001

BM—Body mass; BMI—Body mass index; BMI-z—Body mass index z-score; WC—Waist circumference; WHtR—Waist-to-height ratio; DXA—Dual-energy X-ray absorptiometry; FM—Fat mass; %FM—Percentage fat mass; FFM—Fat-free mass; FLEX—Flexibility; HGR/HGL—Handgrip dynamometry, right and left; ABD—Abdominal resistance; CRF_rel_—Cardiorespiratory fitness, relative; CRF_abs_—Cardiorespiratory fitness, absolute; STS_MC_—Motor competence in STS; STS_TIME_—STS execution time.

**Table 2:** clinical evaluation and metabolic profile of adolescents.

Variables	Immediate Return	Three Months of Usual Activities	ES	CI	*p*
HR_rest_	88.8 ± 15.2	80.6 ± 11.3	−0.61	B	0.001
Systolic BP (mmHg)	68.8 ± 9.1	70.4 ± 5.7	0.21	PH	0.341
Diastolic BP (mmHg)	112.8 ± 11.3	113.5 ± 11.2	0.06	T	0.72
25(OH)D (ng/mL)	20.1 ± 5.4	24.2 ± 7.8	0.61	B	<0.001
TC (mg/dL)	132.7 ± 25.7	137.6 ± 21.7	0.21	PH	0.148
Glucose (mg/dL)	75.6 ± 3.8	74.7 ± 3.3	−0.25	PB	0.364
Triglycerides (mg/dL)	74.1 ± 29.6	64.9 ± 23.0	−0.35	PB	0.08

HR_rest_: resting heart rate; BP: blood pressure; 25(OH)D: vitamin D; TC: total cholesterol.

## Data Availability

Data will be made available on request.

## Abbreviations

25(OH)D: vitamin D; BM: body mass; BMI-z: body mass index z-score; CRF: cardiorespiratory fitness; CRF_abs_: absolute; CRF_rel_: relative; FM: fat mass; FFM: fat-free mass; STS: supine-to-stand; MC: motor competence; WC: waist circumference; WHtR: waist-to-height ratio.
